# Integrating evidence-based interventions in clinical settings in Jordan: a policy brief

**DOI:** 10.12688/f1000research.54362.1

**Published:** 2021-09-16

**Authors:** Mohammad Alzaatreh, Obay A. Al-Maraira, Nazih Abu Tabar, Mohammad R. Alsadi, Huthaifah Khrais, Hamzeh Y Abunab, Khaled M. Kasasbeh, Mohammad Almaani, Malek Khalil

**Affiliations:** 1 Prince Al Hussein Bin Abdullah II Academy for Civil Protection, Department of paramedics, Al-Balqa Applied University, Asalt, Amman, 41111, Jordan; 2 Nursing department, Al- Farabi Private College, Jeddah, Saudi Arabia; 3 Jordanian Nursing Council, Amman, Jordan; 4 Faculty of Nursing, Zarqa University, Amman, Jordan; 5 College of Nursing, Isra University, Amman, Jordan; 6 Quality department, Prince Sultan Military Medical City, Riyadh, Saudi Arabia; 7 Department of Nursing, Emirate Health Services, Dubai, United Arab Emirates

**Keywords:** Policy Brief, EBP, Nursing, Healthcare Professionals, Clinical Practice Guidelines, Jordan.

## Abstract

**Background:** Evidence-based nursing practice (EBNP) is considered a major and very important global paradigm shift. Unfortunately, most healthcare providers and researchers embrace the concept of evidence-based practice (EBP) without integrating this concept in clinical settings. The current situation of EBP and new practice guidelines utilization in Jordan are scarce. This policy brief aimed to discusses the process of utilizing nursing EBP in clinical settings in Jordan.

**Methods:** The authors adopted an action plan utilizing a systematic approach to develop and implement specific strategies and policies to integrate EBP in clinical settings in Jordanian hospitals.

We present an experience of one country in terms of introducing a policy brief to establish an EBP policy accompanied by developing an EBP unit in the hospital's country.

**Results:** A comprehensive description of this policy is provided with reference to the eminent steps of policy analysis and evaluation. In fact, EBP policies and clinical practice guidelines should keep a live document and revise regularly or as needed. Overall, the authors suggest the development of a unit for EBP to deal with issues related to clinical practice guidelines.

**Conclusions:** Expected outcomes for the introduction of the EBP unit and its policy include increase research utilization and accelerated adoption of new evidence, increase the quality of care provided, increase patient, staff, and managers satisfaction, and decrease staff workload by reducing complications associated with medical errors and outdated interventions.

## Introduction

Evidence-based practice (EBP), evidence-based medicine (EBM), or evidence-based nursing practice (EBNP) are all substitutes to what is known about providing medical care or taking a medical decision using the best available knowledge or research findings. ^1^ EBP is considered a major and fundamental global paradigm shift. ^2^ Generally, EBP is generated by identifying the problem, searching alternatives, evaluating the options, and therefore generating the evidence based on available experimental research and linking it to the practice and policy domain to ultimately improve the quality of healthcare and patients outcomes. ^3, 4^ The term EBP is widely used throughout all the healthcare disciplines; such as nursing, medicine, physical therapy, and other fields. ^5^ EBP helped interdisciplinary healthcare teams towards achieving better healthcare outcomes. ^4^ Unfortunately, the utilization of EBP is limited. Most healthcare providers and researchers embrace the concept of EBP without actual integration in clinical environment. ^4^ Also, there is a gap in nurses’ knowledge of the research steps and their abilities in utilizing EBP in the clinical environment. So, this requires innovative solutions beyond the traditional ways of education. ^6^

Personal and system-level challenges in the implementation of EBP contributed to the persistent research to practice gap in healthcare delivery. ^7^ Research utilization (RU) into EBP is struggling with many barriers. One major barrier is that nurses, in general, lack the interest, time, and/or motivation to be involved in the research process due to its complexity. ^8^ Additionally, nurses are ill-prepared at the undergraduate level to understand, conduct, appraise, and consume research results. ^9, 10^ Hence, it’s not possible for nurses who lack the fundamentals skill of research to conduct such a complicated task. ^11^ Nurses will not identify a clinical problem and access a medical database searching for alternative medical options targeting the main concern. After that, nurses should critically evaluate the result for any evidence based on its feasibility, appropriateness, and effectiveness and ultimately generate evidences. ^4^ For these reasons, healthcare organizations and nursing associations should be responsible for connecting or collecting experts who can collect, evaluate, and generate evidence, and reflect it in the form of protocols, practice guidelines, and ultimately regular policies.

In Jordan, the problem with the current situation of EBP is that utilization of the evidence is limited to a few initiatives through clinical practice, and a few attempts to distribute it through conducting educational lectures, seminars, and journal clubs, without implementing any policy that would organize the integration of newly approved clinical related evidence that promotes and enhances the quality of care.

The key solution to improve adherence to EBP and increase RU is to adopt it at the organizational level rather than relying on individual initiative. ^4^ Otherwise, nurses will continue to rely on tradition, authority, limited clinical experience, and intuition as sources of information, turning a blind eye to the new updates in the field of health sciences and nursing science. This will be reflected directly in the quality of care they provide and the outcome they are aiming for.

The purpose of this policy brief is to introduce a policy about how to implement the updated EBP research findings into clinical practice guidelines, protocols, and procedure manuals. This paper presents a structured process from O’Grady (2020) ^12^ that provides policymakers with the background and the existing status of EBP utilization, necessary to be more familiar with the problem of low utilization of EBP and new guidelines. Also, it discusses the contextual factors such as socio-cultural, economic, and moral concerns, ethical, legal, and political factors, and the target audience for this issue. It then describes the proposed solutions and the expected outcomes. Subsequently, it suggests an implementation methodology ^13^ includes the preparation for launching the EBP unit, incorporating the EBP within the institution's mission statement, EBP operational process, analysis of potential risks, and implementation timeline ^14^ and evaluation method.

## Policy context

### Cultural context

EBP is taught at various courses in nursing curriculum at the undergraduate level and after graduation, with the majority of nurses attending many lectures regarding EBP at hospitals in Jordan. These courses have been aimed at ensuring nurses are fully aware of the importance of EBP. However, EBP is not reflected in their practice because nurses lack the knowledge, skills, and resources required to recognize, appraise, and implement interventions based on new and existing evidence. Moreover, the organizational attempts to promote EBP culture and practice are scarce. ^15^ Indeed, the positive attitudes toward EBP prepared nurses to implement EBP but unfortunately, no actual steps have been taken to integrate it into clinical practice.

### Economical context

EBP is intended to produce the best care with minimal cost. Jordan is categorized as a lower-middle-income country. and the average health expenditure in Jordan is 9.8% of gross domestic product. ^16^ The public sector serves more than 60% of the general population in Jordan. Implementing EBP is associated with decreases in length of hospitalization, reduced postoperative complications and hospital-acquired infections, and thus, a decrease in economic burden for health. Unfortunately, the public sector is lacking attempts to integrate EBP in clinical practice. However, some individual initiatives to adopt EBP from the private sector achieved significant outcomes such as a decrease in hospital-acquired infection rate and increase hand hygiene compliance. As an example, new evidence such as an educational interventional program to increase hand hygiene practices in one private hospital in Jordan was implemented and resulted in decreased hospital-acquired infection by 5.6% and decrease the length of stay by three to six days. ^17^

### Ethical and legal factors

The seriousness of not implementing EBP is considered as significant as the ethical issues attending to its application. While utilizing the EBP, we shall use it judiciously. ^18^ EBP helps to bridge the gap between healthcare providers and quality care, which is considered our ethical responsibility towards patients. ^19^

### Political factors

The concept of EBP is not well accepted by managers and policymakers unless it optimizes outputs and translates into financial benefits and money. ^20^ The language of money and effectiveness is an acceptable means to justify service provision. Some research findings might be legitimized as evidence-based if available research supports a favorable outcome. This approach will have the consequences of continuously neglecting implementation and, therefore, widening the gap between evidence and practice. ^21^

### Stakeholders

The stakeholders for this policy in Jordan comprise the Ministry of Health (MOH), Jordanian Council of Nursing (JNC), and Jordanian Nurses and Midwifery Council (JNMC). All healthcare providers, including nurses, are supposed to participate in policy development and implementation. Besides, the continuing education department and quality assurance units should be involved in the process for follow-ups on implementing the policies in the hospitals. Nurse educators and clinical nurse specialists should educate nurses and nursing students about the research process’s role in identifying the problem and proposing appropriate solutions. Health accreditation bodies such as the HealthCare Accreditation Council (HCAC) in Jordan should play a major role in promoting policy implementation in all healthcare settings to enhance quality.

### Issue statement, policy goal, and objectives

The policy issue statement is to develop a method to utilize EBP results in developing clinical practice guidelines into the nursing practice milieu in Jordan.

The policy goal and objectives are first to establish and implement specific strategies and policies to enable professional nurses to provide the best nursing care in Jordan based on clinical practice guidelines derived from the best available evidence. The second is to develop educational and training programs for all professional nurses regarding the significance of embracing the concept of EBP in current clinical practice. Lastly, to establish a reviewing process using a common standard for appraisal of the evidence that is carried out regularly.

## Evaluation of policy options and alternatives

Evaluation criteria for the policy and each alternative must reflect the potential to achieve the policy goals and objectives, decrease cost, consideration of different populations, political, social administrative-technical feasibility, and sustainability. The evaluation process will be based on modified Kraft and Furlong's (2019) evaluation criteria of policy options ^22^ as described in Table 1.

**Table 1.** Description of evaluation criteria, based on Kraft and Furlong (2019).

No	Criteria	Description
1	Effectiveness	What is the likelihood of achieving policy goal and objectives or demonstrated achievement of them?
2	Efficiency	Likelihood of achieving program goals in relation to cost, least cost to a given benefits
3	Equity	Fairness in distribution policy benefits across populations and subgroups
4	Liberty	The alternative extends privacy and individual choices
5	Political feasibility	The likelihood that the alternative will be adopted by policymakers
6	Social acceptability	The likelihood that the alternative will be adopted by medical practitioners
7	Administrative and technical feasibility	The likelihood that the health institutions can implement the policy
8	Sustainability	The likelihood that the alternative can meet current and future demands

### Alternative one: Increase RU

This is an incremental approach for change to increase RU by focusing on presenting the best evidence by identifying, synthesizing, evaluating, and disseminating evidence to professional nurses. This is mainly a personal initiative rather than an organizational one. Such an approach includes journals club (a meeting that involves a group of people discussing articles related to critical topic), better access to the electronic database, medical educational courses, and related workshops. Although it might be a good approach toward better RU, its a personal initiatives that don’t guarantee the sustainability of this evidence. Also, it needs advanced skills in appraising evidence. ^23^ However, this alternative might achieve policy goals. The application of this alternative will indirectly lower the cost while getting the benefit of implementing this policy. This alternative can also be politically feasible, but it is more difficult to achieve. This alternative is also administratively acceptable as it includes minor changes in the current situation of RU. Furthermore, this alternative is socially acceptable as it protects healthcare professionals and will ensure that the patients are receiving optimal care.

### Alternative Two: Establishing a unit responsible for collecting evidence and developing guidelines according to the EBP

Transforming integrated EBP into routine clinical nursing practice is an organizational effort and needs to be supervised by the managerial board and specialized unit or a committee. ^4^ Therefore, developing a policy that acts as a pathway for academics and clinicians, particularly at the bedside, who are usually facing clinical problems to propose and legitimize any new evidence is a means to solve this problem. This alternative will regulate the process of selecting any new evidence or guidelines and describes the process of endorsing it to the EBP unit that is responsible for appraising and integrating the newly adopted practice in the policy and practice manual, and the process of disseminating it.

The EBP unit should include members mainly from nursing as they constitute the vast majority of medical practitioners and also, a representative from other disciplines such as medicine, physiotherapy, pharmacy, laboratory, and respiratory therapy. Also, it will include a consultant and representative from JNC and JNMC. The criteria for the consultant, who must be an expert in the field of EBP research, including that they must; (1) hold a Ph.D. in nursing or in a medicine-related field, (2) have published at least four articles concerning EBP in peer-reviewed journals, and (3) have served as a reviewer in at least two peer-reviewed journals. The criteria for the members in the EBP unit includes that they must; (1) hold at least a masters-level qualification in a nursing or medicine-related field, (2) have published at least one article concerning EBP in peered review journals, (3) attended at least one course related to research ethics, and (4) have at least one year experience in clinical practice. A steering committee should be designed to recruit members for the EBP unit. An official announcement for the EBP unit vacancies should be circulated internally and posted on the website of the institution of implementation. Primarily, this committee will be developed to set the guidelines of the healthcare practices at hospitals based on what constitutes a legitimate source of evidence for healthcare practices. Setting the unit operational bylaws, conducting educational sessions, awareness sessions for hospital employees, clients, and stakeholders, and selecting a model for evidence-based decision making will be also the functions of the EBP unit.

This option can achieve the goal of a new policy and is socially acceptable. Implementing this alternative will indirectly lower the cost of compensation for healthcare professionals and patient health costs by decreasing medical errors and ensuring the quality of care that outweighs the cost of policy application. Further, this option will ensure sustainability. However, it is politically infeasible as this is challenging to achieve because it needs significant and frequent changes in medical procedures that medical practitioners used to do.

### Summary of the comparison

Table 2 presents the final results of the comparison between the alternatives. The evaluation tool consists of eight criteria; the authors evaluated each criterion using a Likert scale ranging from 1-5. (Score 1 indicates very low likelihood, 2 – low likelihood, 3 – moderate likelihood, 4 – high likelihood, 5 – very high likelihood). As the authors of this paper represent different sectors including academic, clinical, and administrative sectors, and hold a Ph.D or are a Ph.D. student in nursing with at least three years of clinical experience, they were responsible for the evaluation. They were requested to independently rate each alternative and to submit it to the primary investigator. Then the primary investigator reviewed all the submitted comparisons and calculated the mean scores displayed in Table 2. The results indicate that the alternative of developing the EBP unit obtained the highest score.

**Table 2.** Evaluation of the alternative options, including total score for each alternative and rank according to the total score.

Criteria	Alternative one	Alternative two
Effectiveness	3	5
Efficiency or coast	3	5
Equity or fairness	4	4
Liberty	3	3
Political feasibility	3	2
Social acceptability	4	3
Administrative feasibility	4	4
Sustainability	3	5
**Total**	27	31
**Ranks**	2	1

### Recommended solution

The authors of this paper suggest the development of a unit for EBP as described in alternative Two. This unit has the main duties of identifying clinical problems and clinical practices utilized to solve the problems, in addition to the clinical practice protocols and guidelines used during routine clinical nursing practice within the hospitals to treat patients’ conditions. However, medical practitioners are encouraged to participate in this process by identifying the clinical problem and searching the literature for any evidence-based practice or guidelines. They are developing a patient, intervention, comparison, outcome, and time (PICOT) ^1^ question to ensure they are identifying the clinical problem. The P includes types of patients/patient populations. Consideration should include sex, ethnicity, and patients with particular healthcare problems. We include interventions or specific methods or treatments of interest. C is a comparison of alternatives in treatment or interventions to the problem. This may also include new or alternative ways to achieve the same outcome. The O is looking at the desired outcome. This must be precise and brief when developing your question. The T is for timing. This can be optional but may be relevant to the particular clinical question. ^1, 20^ Next, they should endorse it officially to the EBP unit for evaluation. Evidence-based practice units will facilitate this process by providing clinicians’ scientific resources to search for up-to-date clinical interventions for different patient cases. Then, the EBP unit in cooperation with related hospital administrators should critically evaluate the outcomes of such interventions in terms of feasibility, appropriateness, meaningfulness, effectiveness, patient satisfaction, and quality of care. Furthermore, EBP unit members will conduct regular meetings with clinical teams and field experts to verify generated evidence. Ultimately, once proven successful against the identified criteria, newly generated evidence and related practices should be reflected in protocols, policies, and practice guidelines and officially disseminated to all related hospital departments and teams.

Expected outcomes for introducing the EBP unit and its policy include positive reflections on administrative, clinical, financial, staff retention, and patient satisfaction. The key performance indicators (KPIs) include; positive patient experience in receiving healthcare services, top management satisfaction, enhanced employee satisfaction and retention, reduction of waste of the hospital resources, decreased length of stay, reduced postoperative complications, and hospital-acquired infections which are where we expect to get the better number after the introduction of the policy and unit. On the other hand, “Magnet organizations have an ethical and professional responsibility to contribute to patient care, the organization, and the profession in terms of new knowledge, innovations, and improvements”. ^24^ Magnet accreditation helps nurses be more involved in the process of decision-making. They can participate in committees such as the EBP committee. Participating in such a committee will enhance the professional growth and improved patient outcomes. ^25^

## Policy implementation

In order to execute the introduction of an EBP policy and unit, a proposed methodology is presented in this section. It includes the adopted theoretical framework for implementation, the preparation for launching the EBP policy and unit, incorporating the EBP within the hospital’s mission statement, EBP operational process, analysis of potential risks, and implementation timeline.

To initiate the proposed change, the process should be started by inviting the selected members of the EBP unit committee for a meeting. The first meeting agenda will focus on introducing the idea to the selected members, getting their buy-in and approval to participate after presenting the current status highlighting the problems and issues faced with EBP, the proposed change, plan of implementation, proposed budget, evaluation of outcomes plan, and potential gains and benefits that will be achieved in introducing the EBP unit.

After the collective approval of the unit members, the EBP unit will be officially launched and announced to the hospital employees. As described by Kouzes and Posner (2012) this kick-start meeting should “model the way” by the presented plans, and “inspire a shared vision” with all unit committee members and “encourage the hearts” to get the change approved, supported, and motivated toward success. ^26^

One of the important aspects of the staff member complies with EBP guidelines can be because EBP unit decisions will affect the whole inter-professional process of providing healthcare. This change can be viewed as a loss of authority on decision making, but it can be mitigated through the suggested awareness sessions and inviting all related team members to participate in journal clubs. ^27, 28^

Lastly, to ensure that the implementation will be incremental, the timeline for project activities is suggested to introduce the project ( Table 3). Actual activities will pass over the six phases as follows: approval for establishing of this policy and unit, launching event, committee meeting and assigning coordinator, awareness sessions, piloting for one use case, and hospital-wide implementation. It is expected that the pilot phase will be conducted within a specified period, while the hospital-wide implementation process will last for about three months as outlined in Table 3.

**Table 3.** Planned timeline for EBP unit implementation activities with expected days and owner of each phase.

Phase	Activity	Owner	Expected working (Mon-Fri) days
Phase-1	Approval to establish EBP unit from the board of directors	Steering committee	1
Phase-2	Recruiting members, consultants, and launching event	Steering committee	20
Phase-3	Committee meeting and assigning a coordinator	EBP chair and members	2
Phase-4	Awareness sessions	EBP coordinator and committee	20
Phase-5	Piloting for one use case	EBP unit and ICU manager	40
Phase-6	Hospital-wide implementation	EBP unit and department managers	180

## Plan for evaluation

To evaluate the outcomes of the policy, the authors suggest developing groups of KPIs to measure the implementation. Pre- and post-implementation of EBP unit measures will be appraised. However, there are four main domains of KPIs: administrative-related KPIs, clinical-related KPIs, patient satisfaction KPIs, and financial-related KPIs.

Examples of administrative-related KPIs are the workload reports (i.e., the number of patients seen by a provider, number of procedures performed), hospital management satisfaction, and healthcare providers’ satisfaction in utilizing the best available evidence for clinical purposes, and percentage of waste reduction in terms of resources. Examples of clinical-related KPIs have included patients’ length of stay in the hospital, numbers of reported medication errors, and percentage of post-treatment complications. Also, by using surveys and questionnaires, patients’ satisfaction with the average time between admission and diagnosis, and the average time between diagnosis and choosing the best medical treatments will be measured. At last, examples of financial-related KPIs include the cost-effectiveness of implementing EBP, periodical net profit, and budget variance. However, the retrieved data from previous KPIs must be analyzed and presented regularly to top management.

The expected positive outcomes of the EBP unit will enhance the adoption and sustainability of the EBP functions. Celebrating the achievements (i.e., improved KPIs scores) would be another strategy for sustainability and enhancing the spirit of teamwork, the sense of achievement among healthcare team members. Besides, this alternative is subjected to modifications based on regular policy evaluation.

## Implication for nursing and health policy

EBP is the keystone of nursing, but marketplace changes call for more collaboration to implement EBP. Current staff can become EBP champions and role models for newer nurses to implement a shared vision for implanting EBP across disciplines. As nurses, we receive many medical questions from the new nurses regarding the best intervention and patients and their families regarding their conditions. Some of these questions we cannot answer without examining the literature and researching the key components necessary to help us, our patients, and patients’ families make sound decisions. Using new evidence to underpin practice without assessing its effectiveness is shortsighted. Nursing colleges and universities are obligated to encourage student nurses to embrace and implement evidence-based practice from the onset of their clinical practice.

## Conclusion

This paper presented an experience of one country, Jordan, regarding policy issue analysis to establish an EBP policy accompanied by developing a unit in the hospital’s country. The paper presented background information about the significance of introducing EBP in providing routine patient care, and then it goes through a comprehensive assessment of the current situations. The paper then moves to describe the change initiative, plan, and methods of implementing EBP along with an evaluation plan and sustainability strategies. EBP is proven to be the need to achieve the set mission and vision to excel as leaders at the local and regional levels of healthcare.

## Data availability

All data underlying the results are available as part of the article and no additional source data are required.
