# Supplementary material for: Integrating evidence-based interventions in clinical settings in Jordan: a policy brief
Source: F1000Res. 2021 Sep 16;10:933. [Version 1] doi: 10.12688/f1000research.54362.1 (PMC8689402; doi:10.12688/f1000research.54362.1)
Supplement: Peer review history

# Peer review: Reviewer response for version 1

**DOI:** 10.5256/f1000research.57842.r96863

**Published:** 2021-12-20

**Authors:** Latefa Ali Dardas

**Affiliations:** 1 School of Nursing, The University of Jordan, Amman, Jordan

This work presents a significant and timely addition to the literature and seems to have promising implications to enhance the quality of health practices. The authors have proposed an action plan utilizing a systematic approach to guide the process of integrating evidence-based practice (EBP) in clinical settings in Jordanian hospitals, and I cannot agree more with authors that most healthcare providers and researchers embrace the concept of EBP without actual integration in clinical environment.

 Within the Jordanian context, it became evident that there is a significant and dramatic increase in the number of published research studies. Nevertheless, minimal changes in practice have been seen in response to these published findings. It is therefore important to tap issues that hinder the integration of EBPs in the clinical setting.

 The authors have chosen a very important and influential factor in this process, which is the lack of biding policies that can guide how to implement the updated EBP research findings into clinical practice guidelines, protocols, and procedure manuals. In doing so, authors provided a comprehensive overview of the policy and the context of its implementation in a way that is accessible to a general reader.

 Authors provided interesting contextual information related to the ‘cultural’, ‘economical’, ‘ethical’, ‘legal’, and ‘political’ aspects in Jordan, highlighting the unique description of each and building upon to propose solutions and alternatives.

 The provided alternatives were made in a way that reflects the potential to achieve the policy goals and objectives, decrease cost, consideration of different populations, political, social administrative-technical feasibility, and sustainability.

 Recommendations made are clear, balanced, and justified on the basis of the presented arguments.

 Overall, this article includes novel and significant information that can be useful in guiding efforts to promote and support an evidence-based practice.

Does the paper provide a comprehensive overview of the policy and the context of its implementation in a way which is accessible to a general reader?

Yes

Is the discussion on the implications clearly and accurately presented and does it cite the current literature?

Yes

Are the recommendations made clear, balanced, and justified on the basis of the presented arguments?

Yes

Reviewer Expertise:

Mental Health

I confirm that I have read this submission and believe that I have an appropriate level of expertise to confirm that it is of an acceptable scientific standard.

# Peer review: Reviewer response for version 1

**DOI:** 10.5256/f1000research.57842.r99178

**Published:** 2021-12-02

**Authors:** Tonje Lossius Husum

**Affiliations:** 1 Centre for Medical Ethics, Institute for Health and Society, University of Oslo, Oslo, Norway; 2 Oslo Metropolitan University, Oslo, Norway

Thank you for the opportunity to review this article. The article is about developing an action plan utilizing a systematic approach to develop and implement specific strategies and policies to integrate EBP in clinical settings in Jordanian hospitals. The topic is an important topic in contemporary times, and It’s an important task to do research on how to implement EBP in clinical settings. 

 The concept of evidence-based care is not new, but I think that all the questions and the science about how to implement EBP in clinical settings has been an underdeveloped and underprioritized area. This research contributed with new important and concrete knowledge about how it is possible to work with implementing EBP in a clinical setting.

 This study is conducted in a somatic hospital setting. To work according to and to implement EBP in clinical settings is also an important matter in mental health care. In the future I hope it will be more focus and scientific development in this area to.

 The abstract is informative and well written, so is the paper itself. It has good structure and is easy to follow.

 The authors could maybe also add something about ethical challenges when implementing EBP, and something about inhibitors and promoters in implementing the suggested plan for EBP. Also, the authors could maybe add a risk analysis in implementing the suggested model.

 Altogether an important work and scientific development concerning implementing EBP in clinical settings which hopefully will inspire other research groups to do research in the topic of implementation science.

Reviewer Expertise:

Ethics in mental health care and medical ethics in general.

I confirm that I have read this submission and believe that I have an appropriate level of expertise to confirm that it is of an acceptable scientific standard.

# Response

**Published:** 2021-12-04

**Authors:** mohammad mohammad

**Affiliations:** albaqa applied university, Jordan

Dear Dr. Tonje Lossius

 Thanks for the time and effort you spent in reviewing this paper.

 Also, thanks for your comments.

 In the revised version, I will include one more step after approving any evidence that is obtaining ethical approval. 

 The next step after this brief is the actual implementation. We are planning to pilot this brief in one educational hospital.
